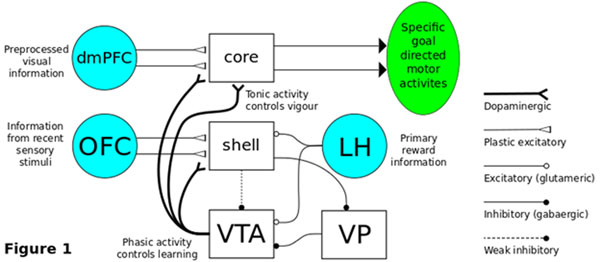# A limbic system model for the control of release of tonic dopamine and its effect on response vigor

**DOI:** 10.1186/1471-2202-12-S1-P256

**Published:** 2011-07-18

**Authors:** Graeme Hattan, Bernd Porr

**Affiliations:** 1 School of Engineering, University of Glasgow, Glasgow, G12 8LT, UK

## 

It has been proposed that phasic dopamine activity is responsible for learning whereas tonic dopamine is rather responsible for the response vigour [1]. In contrast to previous more abstract work on this topic we have created a biologically realistic limbic system model where the release of tonic dopamine controls the overall response vigour of a simulated agent and where the release of phasic dopamine controls learning. Tonic dopamine provides a signal similar to the average rate of reward suggested by Daw [1] and directly affects the response rate of the agent.

Here, we show how tonic dopamine is controlled by the sub-cortical nuclei of the limbic system, especially by the nucleus accumbens (NAc) shell and the ventral pallidum [2]. By embedding these nuclei in a system level model of the limbic system we are able to generate tonic activity as seen in behavioural experiments. The NAc shell thereby learns new associations between rewards and stimuli which are then used to control the tonic dopamine which in turn controls the vigour. The learning which takes place in the shell is triggered by phasic dopamine and is implemented as a third factor [3].

To demonstrate our model, we have set up a simple food seeking task [3] where an agent has to learn to associate the colour of a food pot with a reward contained inside. The VTA activity is initially controlled by the unexpected rewards received, which generate phasic activity and learning. Learning in the NAc core allows the association of specific visual information with specific motor activities. Learning in the NAc shell allows specific sensory information to disinhibit the VTA via a shell - ventral pallido pathway (Figure 1). The subsequent increase in tonic dopamine activity allows for an increase in vigour brought about purely by the presentation of reward predicting stimuli.

**Figure 1:**